# Plant Growth and Drought Tolerance-Promoting Bacterium for Bioremediation of Paraquat Pesticide Residues in Agriculture Soils

**DOI:** 10.3389/fmicb.2021.604662

**Published:** 2021-03-18

**Authors:** Phatcharida Inthama, Pamon Pumas, Jeeraporn Pekkoh, Wasu Pathom-aree, Chayakorn Pumas

**Affiliations:** ^1^PhD Degree Program in Environmental Science, Environmental Science Research Center, Faculty of Science, Chiang Mai University, Chiang Mai, Thailand; ^2^Department of Environmental Science, Faculty of Science and Technology, Chiang Mai Rajabhat University, Chiang Mai, Thailand; ^3^Department of Biology, Faculty of Science, Chiang Mai University, Chiang Mai, Thailand; ^4^Research Center in Bioresources for Agriculture, Industry and Medicine, Department of Biology, Faculty of Science, Chiang Mai University, Chiang Mai, Thailand

**Keywords:** paraquat, plant growth-promoting, bioremediation, soil, pot experiment, contaminated agricultural soil

## Abstract

Thailand is an agricultural country. However, agricultural productivity relies on the heavy use of herbicides, especially paraquat. Paraquat accumulation is emerging as a problem in an ever-growing portion of agricultural land. Paraquat residues are toxic to plants, animals, and aquatic organisms in the environment. Biological remediation is a process that can mitigate agricultural chemical contaminants. One of the interesting bioremediators is bacteria. Not only do certain soil bacteria remediate paraquat, but some of them also possess plant growth-promoting properties, which provide advantages in field application. Thus, this study aimed to screen soil bacteria that could degrade paraquat and, at the same time, promote plant growth. Bacteria were isolated from paraquat-treated agricultural soil in Mueang Kaen Pattana municipality, Chiang Mai province, Thailand. On the basis of morphological and 16S rDNA sequence analyses, the selected bacterium was identified as *Bacillus aryabhattai* strain MoB09. It is capable of growing in nitrogen-free media. *B. aryabhattai* growth and paraquat degradation were found to be optimum at pH 7 and 30°C. This selected strain also possessed plant growth-promoting abilities, including indole production, siderophore production, phosphate solubilization, and 1-aminocyclopropane-1-carboxylic acid deaminase activity. Paraquat degradation was also evaluated in pot experiments of cowpea (*Vigna unguiculata*). It was found that this strain could remediate the paraquat residue in both sterilized and non-sterilized soils. The cowpea plants grown in paraquat-contaminated soil with *B. aryabhattai* showed longer root and shoot lengths than those grown in soil without bacterial inoculation. In addition, *B. aryabhattai* also promoted the growth of cowpea under induced drought stress. These results suggested that *B. aryabhattai* could be applied to mitigate paraquat residue in soil and also to promote plant productivity for the organic crop production.

## Introduction

Vegetables have long been consumed worldwide, and the trend for vegetable consumption as clean and healthy food is currently increasing. However, vegetable production relies heavily on the use of pesticides for crop protection, increase in production yields, and product quality improvement as well as product appearance. Cowpea (*Vigna unguiculata*) is an annual herbaceous legume from the genus *Vigna*. Due to its tolerance for sandy soil and low rainfall, it is an important crop in the semiarid regions across Africa and Asia. In addition, cowpea has been reported to be highly sensitive to water and herbicide stress (Oyewole et al., 2017; Silva et al., 2020).

The global uses of herbicides, chemical compounds that are toxic to plants and aim for the elimination of unwanted weeds, have been increasing significantly over time (Gianessi, 2013). Paraquat (1,1-dimethyl-4,4-bipyridyl dichloride) is a herbicide that has been widely used in agriculture for more than 60 years, and but at the present, it has been banned or disallowed in many countries (Bromilow, 2004). It is a non-selective herbicide that diverges electron flow from the photosystem, resulting in the production of lethally reactive free radicals, superoxide radicals, which damage plant tissues (Reczek et al., 2017). Paraquat may be classified as immobile herbicides according to its strong adsorption on soil particles. However, inappropriate use of paraquat, for instance, repeated and excessive (beyond the recommended concentration) use, over the years may cause an accumulation of paraquat in the environment. Environmental contaminations of paraquat have been reported over the world, for example, Indonesia (Ardiwinata et al., 2019), Brazil (Veríssimo et al., 2018), and Thailand (Teerakun et al., 2020). Although the concentrations of paraquat contamination in some areas are still below the tolerance limit, long-term exposure to those paraquat residues might result in harmful effects on humans and mammals and the food chain (Huang et al., 2019).

Several processes have been reported for a removal of those residues, e.g., biodegradation, photodegradation, oxidation, flocculation, filtration, and adsorption. However, biological remediation, the so-called biodegradation, is a more economical and environment-friendly approach. Although naturally occurring soil microbes can alter those residual chemicals, this process generally takes time to achieve. Depending upon the texture and soil composition, natural microorganisms can only remediate less than 1% of residual paraquat in the soil (Roberts et al., 2002). Thus, it is necessary to enhance paraquat-degrading microorganisms for restoration of paraquat-contaminated sites. Many bacteria capable of degrading pesticides have been isolated and identified such as *Bacillus*, *Pseudomonas*, *Flavobacterium*, *Arthrobacter*, *Diaphorobacter*, *Klebsiella*, *Ochrobactrum*, *Agrobacterium*, and others (Ataikiru et al., 2020).

Microorganisms also perform an important role in supplying nutrients to plants and reducing the demand of chemical fertilizers (Cakmakci et al., 2006). It is commonly known that microorganisms are capable of plant growth regulator productions such as auxins, gibberellins, siderophores, phosphate-solubilizing substances (Khalid et al., 2009), and 1-aminocyclopropane-1-carboxylic acid (ACC) deaminase activity (Penrose and Glick, 2003). In addition, the use of bacteria as inoculants simultaneously increased phosphate uptake by the plant and increased crop yields. Many bacterial strains from the genera *Pseudomonas*, *Bacillus*, *Rhizobium*, *Burkholderia*, *Achromobacter*, *Agrobacterium*, *Micrococcus*, *Enterobacter*, *Flavobacterium*, and *Erwinia* have been known as the producer of the powerful phosphate solubilizers (Esitken et al., 2003). The mechanism for mineral phosphate solubilization is the production of organic acids and acid phosphatases. Indole-3-acetic acid (IAA) is one of the most physiologically active plant growth regulators produced from L-tryptophan metabolism by soil fungi and bacteria (Muhammad and Frankenberger, 1991). Siderophores are the important compounds for the survival and growth of bacteria in the soil and in aqueous environments. It has been reported that siderophore mediated iron transport found mostly in Gram-negative bacteria and also in Gram-positive bacteria such as *Bacillus*, *Staphylococcus*, and *Streptomyces*. However, siderophores and their substituted derivative structures varied from one species to another (Kannahi and Senbagam, 2014). Besides, it was found that *Pantoea ananatis*, *Pseudomonas putida*, *Brevibacillus agri*, *Bacillus subtilis*, and *Bacillus megaterium* were growth boosters for the plant (endophyte) as well as the area of rooting (rhizosphere). Some *Bacillus* species also possess both plant growth-regulating and herbicide-degrading properties. For example, *Bacillus* sp. ACD-9 could degrade acetochlor and show phosphate-solubilizing activity on maize seedlings (Li et al., 2020).

Other than pollutants remaining in soil, environmental factors could also generate various stresses, which affect plant growth and development. Environmental factors such as drought and salinity are of interest. Their effects on crop yield and quality for many plants have been reported (Salehi-Lisar and Bakhshayeshan-Agdam, 2016). Interestingly, some bacteria showed dual-plant-benefit properties, both growth promotion and stress protection, such as *Bacillus fortis* strain SSB21, which could promote plant growth and contains osmolyte production to improve salinity tolerance in *Capsicum annum* L. (Yasin et al., 2018). *Bacillus methylotrophicus* CSY-F1 produces plant growth-promoting substances and alleviates drought stress in cucumber (*Cucumis sativus*) grown in soil with high ferulic acid (Hou et al., 2018). *Bacillus aryabhattai* SRB02 could promote the growth and heat resistance of soybean by production of phytohormones (Park et al., 2017).

These described bacteria could play vital roles as bio-fertilizers, bio-stimulants, bio-protectants, and biodegradators (Andriani et al., 2017). However, to our knowledge, multi-plant growth-promoting, drought-protective, and paraquat-degrading abilities from individual bacteria have yet been observed. Thus, this study aims for the discovery of a reasonable potential strain and its optimal culture condition, which could be acclimatized to the target polluted sites and enhance the bioremediation process and increase crop production.

## Materials and Methods

### Chemicals Used

Paraquat dichloride hydrate standard was obtained from Sigma-Aldrich, Merck, Germany. Solvents for HPLC were purchased from SM Chemical Supplies Co., Ltd. All chemicals were of the highest purity that is commercially available.

### Isolation and Identification of Paraquat-Degrading Bacteria

Soil specimens were collected from an agricultural area in Mueang Kaen Pattana municipality, Chiang Mai province, Thailand. This area is under heavy chemical use, including paraquat. One kilogram of the soil samples was collected from the first 10–15 cm depth, pooled, and sieved. Samples were air-dried and stored in sterile plastic bags at 4°C until use. The paraquat-degrading bacteria were screened for molybdenum-reducing ability and for paraquat utilization capacity.

#### Screening by Molybdenum-Reducing Method

Ten grams of the soil samples were firstly suspended in sterile tap water, and 0.1 ml of the suspended soil was transferred to low phosphate molybdate agar (LPMA) (pH 7.0) containing glucose (1%), (NH_4_)_2_SO_4_ (0.3%), MgSO_4_⋅7H_2_O (0.05%), NaCl (0.5%), yeast extract (0.05%), Na_2_MoO_4_⋅2H_2_O (0.242%) Na_2_HPO_4_ (0.05%), and agar (1.5%) (Yunus et al., 2009) and further incubated at room temperature for 48 h. The presence of blue colonies indicates the presence of molybdenum-reducing bacteria, which might have paraquat-degrading properties. The intense blue colonies were repeatedly streaked on low phosphate media (LPM) agar to obtain the axenic bacterial culture. Molybdenum-reducing bacterial strains were collected for further colony identification.

#### Screening by Enrichment Method

For the isolation of bacteria using paraquat as the sole carbon and energy source, mineral salt medium (MSM) (pH 7.0), consisting of 2 g of (NH_4_)_2_SO_4_ (0.2%), KH_2_PO_4_ (0.15%), Na_2_HPO_4_ (0.15%), MgSO_4_⋅7H_2_O (0.02%), CaCl_2_⋅2H_2_O (0.001%), and FeSO_4_⋅7H_2_O (0.0001%), was used (Singh and Walker, 2006). Paraquat was added in MSM for enrichment. Two grams of each soil sample was added to 20 ml of MSM containing 0.5 g/L paraquat and incubated in the dark at 30°C under a shaking condition (150 rpm), for 7 days. Five milliliters of these suspensions was then transferred to fresh MSM containing 1 g/L paraquat and further incubated for 7 days. The plates were incubated at 37°C for 24 h. Colonies were collected for further colony purification.

### Screening for Paraquat Degradation of the Isolated Bacterial Strains

Paraquat degradation of the isolated bacterial strains was performed in a 250-ml tube containing 100 ml of nutrient broth (NB medium) (pH 7.0), consisting of peptone (0.5%), yeast extract (0.3%), and 0.5% NaCl. The tubes were then incubated for 7 days, under continuous stirring on a rotary shaker at 150 rpm. Bacterial growth was followed by taking a sample of 2 ml of cultures after every 24 h until 72 h of incubation. The paraquat concentration was evaluated spectrophotometrically based on the AOAC Official Method 969.09 (Tsai and Chen, 2013). One milliliter of each standard concertation and sample was mixed with 1 ml of sodium dithionite (0.04%) in 1 M NaOH; then the mixture was diluted to 10 ml in a volumetric flask. The absorbance at 600 nm was immediately measured by using a spectrophotometer.

### Plant Growth Promotion of the Isolated Bacterial Strains

The isolated bacteria were cultured for the measurement of plant growth-promoting substances such as IAA, siderophore, phosphate solubilization, and ACC deaminase activity.

#### IAA Production

Indole-3-acetic acid production of the isolated bacteria was determined spectrophotometrically as described previously (Lasudee et al., 2018). A 5-mm-diameter agar plug of bacteria-grown nutrient agar was transferred into 5 ml of nutrient broth containing 2 mg/ml of L-tryptophan (Gordon and Weber, 1951). Tubes were incubated at 28 ± 0.2°C with continuous shaking at 110 rpm for 5 days. The supernatant was collected by centrifugation at 11,000 rpm for 5 min. IAA concentration was quantified by mixing 1 ml of the supernatant with 2 ml of Salkowski’s reagent (Glickmann and Dessaux, 1995). The mixer was then incubated in the dark. A pink color indicated IAA production, which was measured for optical density at 530 nm using a spectrophotometer. The IAA concentration was approximated based on a calibration curve of pure IAA standard.

#### Siderophore Production

Siderophore production of all isolate bacteria was detected by chrome azurol S (CAS) assay (Schwyn and Neilands, 1987). A 5-mm-diameter agar plug of bacteria-grown nutrient agar was placed on CAS agar and incubated for 7 days at 28 ± 0.2°C in a dark room. An orange zone around the agar plugs indicated positive siderophore production.

#### Phosphate-Solubilizing Activity

Phosphate-solubilizing activity of all isolate bacteria was measured on Pikovskaya (PVK) agar (Pikovskaya, 1948), containing tricalcium phosphate (0.5%). A 5-mm-diameter agar plug of bacteria-grown nutrient agar was inoculated to PVK agar plates and incubated at 28 ± 0.2°C for 7 days. The visibility of a clear zone around the agar plug showed positive phosphate-solubilizing activity (Nautiyal, 1999).

#### ACC Deaminase Activity

1-aminocyclopropane-1-carboxylic acid deaminase activity was detected by using the method reported by Palaniyandi et al. (2014). The *B. aryabhattai* strain MoB09 was grown on minimal media (Dworkin and Foster, 1958) without a nitrogen source (negative control), minimal media supplemented with ammonium sulfate (positive control), and minimal media supplemented with ACC at a final concentration of 3 mmol/L. All agar plates were incubated at 30°C for 7 days. Growth on the additional ACC medium showed positive ACC deaminase activity.

### Characterization and Identification of the Isolated Bacterial Strains

The selected bacterial isolates were identified by the 16S rRNA gene sequence analysis. Genomic DNA was extracted using a TIANamp Bacteria DNA Kit (Tiangen) according to the manufacturer’s instructions. The 16S rRNA gene from genomic DNA was amplified by polymerase chain reaction (PCR) using bacterial universal primers 27F (5′-AGAGTTTGATCCTGGCTCAG-3′) and 1492R (5′-GGTTACCTTGTTACGACTT-3′). The PCR was carried out in an MJ Mini Personal Thermal Cycler (Bio-Rad) in 50 μl reactions containing 10 μl of 5 × Phusion Green HF buffer (Thermo Scientific), 1 μl of 10 mM DNTP (Bioline), 1.0 μl of 10 mM primer 27f, 1.0 μl of 10 mM primer 1492R, 1.5 μl of DMSO, 0.5 μl of 2 U/μl Phusion Hot Start II DNA polymerase (Thermo Scientific), and approximately 100–250 ng of template DNA. PCR was carried out at an initial denaturation step at 98°C for 30 s, followed by 30 cycles at 98°C for 7 s, 50°C for 20 s, and 72°C for 45 s and a final extension step at 72°C for 7 min. The amplicons were purified with the Universal DNA Purification Kit (Tiangen) and sequenced in both directions by using the same primers. The sequences were compared by aligning the result with the sequences in GenBank using the Basic Local Alignment Search Tool (BLAST) search program at the National Center for Biotech Information (NCBI), with the closely related *Bacillus* strains based on 16S rRNA gene sequences. Phylogenetic analysis was performed based on the neighbor-joining method with MEGA X software. The sequence identity matrix analysis was performed with BioLign software ver. 4.0.6.2 (Lau et al., 2020).

### Optimization of Paraquat Degradation

To optimize paraquat degradation, some important abiotic factors were chosen according to Ouided and Abderrahmane (2013). The optimum pH level was tested at 5.0, 6.0, 7.0, 8.0, and 9.0, and the optimum temperature was observed at 30, 37, and 40°C. All treatments were performed in a 250-ml tube containing 100 ml of NB medium. Utilization of paraquat as a sole carbon source was performed in a 250-ml tube containing 100 ml of MSM, pH 7.0 and 30°C. The tubes were then incubated for 7 days, under continuous stirring on a rotary shaker at 150 rpm. Bacterial growth was followed by taking a sample of 2 ml of cultures after every 24 h until 72 h of incubation. The remaining paraquat was measured by HPLC according to the methods of Wahyu (2013). The HPLC system used for analyzing paraquat was Agilent HPLC 1260 Infinity II with diode array detection (DAD) (Agilent). Analytical column was a C18 column (4.6 × 150 mm) from Vertical. The mobile phase consisted of 5 g of NaCl in 600 ml distilled water, where the pH level was pre-adjusted to 3.0 with HCl, and then the solution was mixed with 400 ml of acetonitrile. Flow rate of the mobile phase was fixed at 1.0 ml/m. DAD wavelength was 257 nm. Twenty microliters each of paraquat standard and sample was injected into the chromatographic column by an autosampler system. Chromatograms were recorded, and the peak area was quantitatively measured with a computer software from the manufacturer.

### Paraquat Degradation and Growth Promotion of Cowpea (*V. unguiculata*)

Paraquat degradation in pot experiments with cowpea (*V. unguiculata*) was conducted in order to examine the effects of bacterial inoculation on plant growth and paraquat degradation. Soil samples (1.5 kg) were spiked with paraquat to a concentration of 200 mg/kg. Cowpea (*V. unguiculata*) was used as a representative of dicotyledon plants. Cowpea (*V. unguiculata*) seeds (Chia Tai Brand, Chia Tai Company Limited, Thailand) were surface sterilized by being immersed sequentially in 2% (v/v) sodium hypochlorite for 1 min, 95% (v/v) ethanol for 1 min, and 70% (v/v) ethanol for 1 min and then washed with sterile distilled water for 1 min; these steps were repeated three times (Lasudee et al., 2018). Five decontaminated seeds were randomly selected to check for surface sterility on nutrient agar. Cell suspension was prepared from the selected strain grown in nutrient broth. Surface-sterilized seeds were mixed with 10^8^ CFU/ml of the bacteria and incubated on a shaker at 120 rpm at 30°C for 16–18 h before being sown. Seedlings were watered once a day with sterile distilled water for 7 days at room temperature. Seven-day-old seedlings with two leaves were transferred to a pot.

The experiment was carried out in a pot (15-cm diameter × 15-cm height) containing 1.5 kg of soil and being kept in a closed room at room temperature for 6 weeks, with five replicates per treatment. Pots were arranged in a completely randomized arrangement in a room. Tap water was supplied once a day to full container capacity and kept at 1 cm above soil level. The following treatments were investigated: (1) sterilized soil with paraquat, (2) sterilized soil with paraquat and seeds mixed with bacterial cell suspension, (3) non-sterilized soil, (4) non-sterilized soil with paraquat, and (5) non-sterilized soil with paraquat and seeds mixed with bacterial cell suspension. At the end of the experiment (6 weeks after sowing), the following growth parameters were recorded: fresh weight (g), dry weight (g), root length (cm), total length (cm), leaf length (cm), and paraquat residue in soil, which was extracted and estimated by LC-MS (In house method, 1997).

### Growth Promotion of Cowpea (*V. unguiculata*) in Drought Conditions

Growth promotion of cowpea (*V. unguiculata*) in drought conditions and under well-watered conditions of the selected strain was performed in a pot experiment using the same protocol as described above. The treatments for drought tolerance promotion included (1) drought-induced treatment, (2) drought-induced treatment with seeds mixed with bacterial cell suspension, (3) well-watered conditions, and (4) well-watered conditions with seeds mixed with bacterial cell suspension. Drought stress was induced by completely withholding water starting on 35 for 10 days. The water deficit treatment was imposed through withholding water from 35 to 45 days after emergence. At the end of the experiment (day 45 after sowing), the maximum water stress was reached after 10 days of treatment; the following growth parameters were recorded: fresh weight (g), dry weight (g), root length (cm), total length (cm), leaf length (cm), chlorophyll content (mg/g dry weight), and proline content (mmol/dry weight). Proline content was determined by the rapid colorimetric method (Bates et al., 1973). Chlorophyll content was determined spectrophotometrically based on the standard method of Arnon (1949).

## Results

### Screening and Identifying the Paraquat-Degrading Bacteria

**FIGURE 1:**
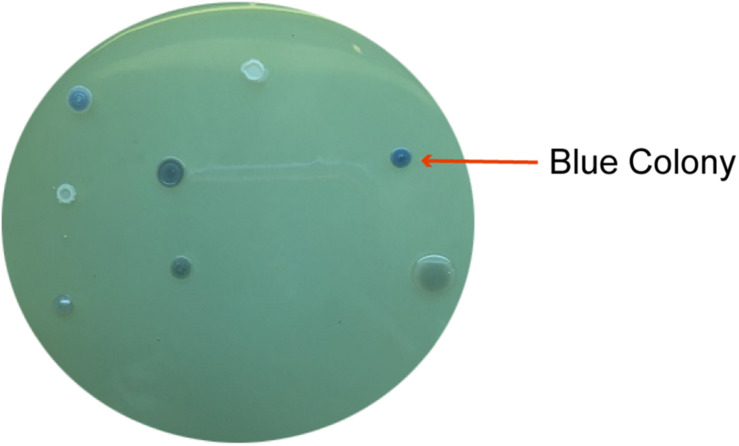
Several blue colonies on LPMA indicated the molybdenum-reducing ability of the isolated bacteria.

According to the variety of bacteria in a soil, it is difficult to isolate desired bacteria from a single method. In this study, paraquat-degrading bacteria were isolated from two methods: molybdenum-reducing ability using LPMA and paraquat utilization capacity using MSM containing paraquat. It was found that 12 morphologically different isolates were selected from LPMA and assigned as MoB strains. The blue colonies on LPMA indicated molybdenum-reducing ability (Figure 1). For screening with MSM containing paraquat, 10 different morphological colonies were selected and assigned as MSM strains. Colony morphology, Gram staining, and light compound microphotographs of those isolates were shown in the Supplementary Material. The 22 selected isolates from both screening methods were then examined for their paraquat degradation.

**FIGURE 2:**
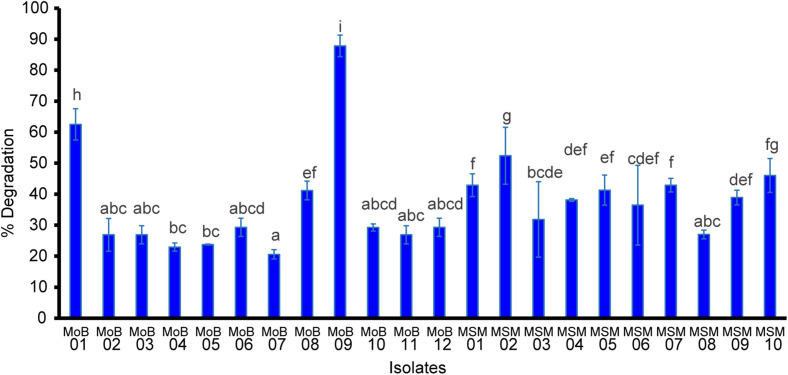
Paraquat degradation of the selected isolates. Data were presented as mean ± SD of three replicas. Letters indicate a significant difference (*p* < 0.05) using ANOVA with *post hoc* analysis.

Figure 2 shows that all the selected isolates could degrade paraquat in a range of approximately 10–45%. The highest paraquat-degrading ability was found in isolate MoB09, which was from the screening by the molybdenum-reducing method, followed by MoB01 and MSM02. A previous study reported that molybdenum-reducing microorganisms showed multiple detoxification capacities; for example, *Klebsiella oxytoca* strain SAW-5 could detoxify Mo and degrade glyphosate (Sabullah et al., 2016). Molybdenum-reducing *Bacillus* sp. strain Zeid 14 and *Burkholderia* sp. strain NENI-11 could utilize acrylamide as a source of electron donor for reduction and were able to grow on media containing acrylamide, acetamide, and acetonitrile (Mansur et al., 2016; Mohd et al., 2016). *Enterobacter* sp. strain Saw-1 and *Bacillus* sp. strain Neni-12 could consume the pesticide coumaphos as an alternative carbon source for growth (Sabullah et al., 2016; Rusnam and Gusmanizar, 2019). However, a previous study found that although some molybdenum-reducing bacteria can successfully utilize pesticides as a carbon source, those pesticides cannot be used as an electron donor for molybdenum reduction (Sabullah et al., 2016). Thus, screening by an enrichment method was also involved in this study. Our result, similar to those of previous studies, revealed that the enrichment method could aid successfully in the screening of xenobiotics-degrading isolates, for example, carbofuran- and paraquat-degrading bacteria (Ataikiru et al., 2020), glyphosate-degrading bacteria (Moneke et al., 2010), chlorpyrifos-degrading bacteria (Parmar et al., 2018), and organophosphorus insecticide diazinon-degrading bacteria (Cycoń et al., 2009). The enrichment method could assist in isolating pesticide-degrading strains and events from a non-pesticide impacted area (Ataikiru et al., 2020). Consequently, screening from various methods may enhance success of the desired potential strains.

### *In Vitro* Plant Growth-Promoting Potential

The *B. aryabhattai* strain MoB09 showed growth-promoting substance production comparable to that of the “Bacillus” group (Table 1). Previous studies reported that bacteria in the “Bacillus” group can produce plant growth-promoting substances. For instance, *B. aryabhattai* SRB02 produced 1.5 ng/100 ml of cytokinin, 2 ng/100 ml of abscisic acid, 2.25 ng/100 ml of gibberellins, and 5.7 μg/ml of IAA and induced growth promotion of soybeans (Park et al., 2017). *B. megaterium* STB1 produced IAA and cytokinins, which can promote the growth of tomatoes (Francisco et al., 2020). *Bacillus amyloliquefaciens* UCMB5113 produced 2.3 ± 0.1 μg/mL of IAA, which can promote *Arabidopsis thaliana* growth by auxin, gibberellin, cytokinin, and brassinosteroid production (Shashidar et al., 2017). *B. amyloliquefaciens* FZB45 produced 2.3 ± 0.1 μg/ml of IAA (Camilo and Joseph, 2010), while *B. amyloliquefaciens* HYD-B17, *Bacillus licheniformis* HYTAPB18, *Bacillus thuringiensis* HYDGRFB19, *B. subtilis* RMPB44 produced 7.2–32.5 g/mg of IAA. *B. aryabhattai* strain MoB09 also tested positive for gibberellin, cytokinin, P solubilization, and siderophore production. MoB09 tested positive for ACC deaminase activity (Supplementary Data). This strain can be grown on an ACC supplied medium, indicating that it can utilize ACC as a nitrogen source (Penrose and Glick, 2003). The ACC deaminase activity of strain MoB09 was added in the Supplementary Figure.

**TABLE 1:** Plant growth-promoting abilities of bacterial isolates.

**Isolates**	**IAA (μg/ml) in NB**	**Siderophore (mm)**	**Phosphate soluble (mm)**
MoB01	131.90 ± 0.68^k^	1.4 ± 0.2^b^	0.8 ± 0.0^a^
MoB02	9.97 ± 0.25^bc^	−	−
MoB03	30.51 ± 0.53^gh^	1.6 ± 0.2^cd^	−
MoB04	177.55 ± 1.32^l^	1.76 ± 0.1^de^	−
MoB05	116.03 ± 1.86^j^	1.43 ± 0.1^bc^	−
MoB06	30.78 ± 0.24^gh^	1.4 ± 0.0^b^	−
MoB07	35.53 ± 1.29^h^	−	1.0 ± 0.0^b^
MoB08	16.37 ± 0.27^cd^	1.8 ± 0.0^de^	−
MoB09	134.75 ± 8.63^k^	3.6 ± 0.1^f^	0.8 ± 0.0^a^
MoB10	21.76 ± 0.22^def^	−	−
MoB11	24.38 ± 0.00^efg^	−	−
MoB12	17.82 ± 0.65^de^	−	−
MSM01	6.81 ± 1.15^ab^	−	−
MSM02	71.39 ± 9.73^i^	1.1 ± 0.0^a^	−
MSM03	27.28 ± 7.31^fg^	1.9 ± 0.1^e^	−
MSM04	3.61 ± 0.74^ab^	1.9 ± 0.0^e^	−
MSM05	4.69 ± 7.31^ab^	1.0 ± 0.0^a^	−
MSM06	2.16 ± 0.74^ab^	−	−
MSM07	1.56 ± 0.92^a^	−	−
MSM08	14.99 ± 1.94^cd^	−	−
MSM09	5.73 ± 2.17^ab^	−	−
MSM10	14.28 ± 0.62^cd^	−	1.00.0^b^

*Data were presented as mean ± SD of three replicas. Letters indicate a significant difference (*p* < 0.05) using ANOVA with *post hoc* analysis.*

**FIGURE 3:**
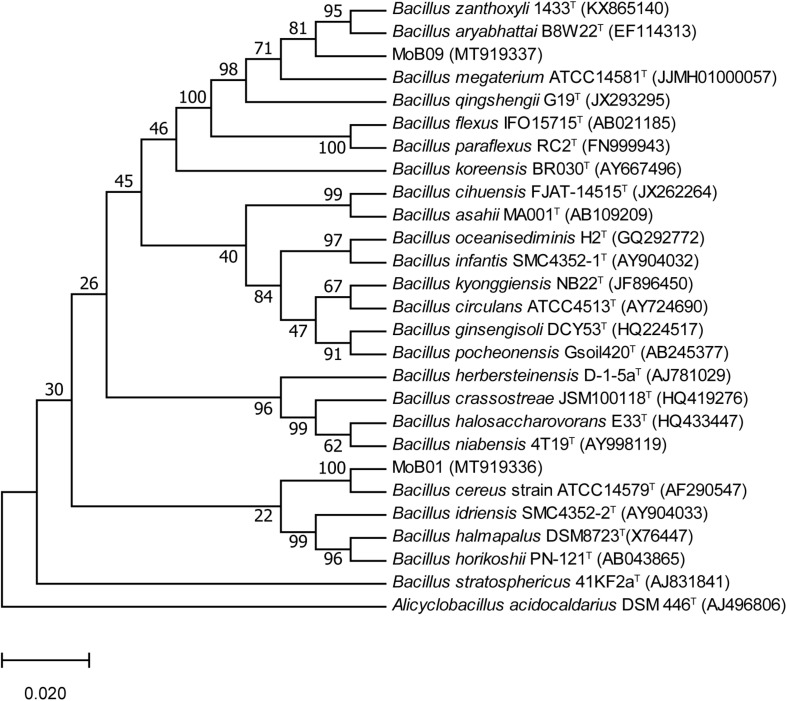
Phylogenetic tree of strains MoB01 and MoB09 with closely related *Bacillus*-type species based on 16S rRNA gene sequences, constructed using the neighbor-joining method. Bootstrap values (expressed as percentages of 1,000 replications) of above 50%.

Almost complete 16S rRNA gene sequences of isolate MoB01 (1,400 bp) and isolate MoB09 (1,414 bp) were obtained and submitted to GenBank^1^ under accession numbers MT919336 and MT919337, respectively. Phylogenetic analysis based on the neighbor-joining method displayed that both MoB01 and MoB09 were clustered in members of the genus *Bacillus* (Figure 3). The sequence identity matrix analysis from BioLign software showed the highest similarity (0.999) of isolate MoB09 to *B. aryabhattai* strain pgB (MK519199), followed by (0.998) *Bacillus zanthoxyli* strain 1910ICU241 (MT225770) and (0.997) *B. megaterium* strain JYW3 (MN161199), respectively. Thus, MoB09 was identified as *B. aryabhattai* strain MoB09. Isolate MoB01 was closely related to *Bacillus cereus* strain ATCC14579 (AF290547), which was reported as an opportunistic pathogen causing food poisoning such as diarrheal or emetic syndromes (Ivanova et al., 2003). Therefore, only isolate MoB09 was selected for further experiments.

*Bacillus aryabhattai* strain MoB09 performed comparable growth-promoting substance production to those of the “Bacillus” group. Previous studies reported that bacteria in the “Bacillus” group can produce plant growth-promoting substances such as *B. aryabhattai* SRB02, which produced 1.5 ng/100 ml of cytokinin, 2 ng/100 ml of abscisic acid, 2.25 ng/100 ml of gibberellins, and 5.7 μg/ml of IAA and promote soybean growth (Park et al., 2017). *B. megaterium* STB1 produced IAA and cytokinins and could promote growth of tomato (Francisco et al., 2020). *B. amyloliquefaciens* UCMB5113 produced 2.3 ± 0.1 μg/ml of IAA. It promoted *A. thaliana* growth by auxin, gibberellin, cytokinin, and brassinosteroid production (Shashidar et al., 2017). *B. amyloliquefaciens* FZB45 produced 2.3 ± 0.1 μg/ml of IAA (Camilo and Joseph, 2010), and *B. amyloliquefaciens* HYD-B17, *B. licheniformis* HYTAPB18, *B. thuringiensis* HYDGRFB19, and *B. subtilis* RMPB44 produced 7.2–32.5 g/mg protein of IAA. They were also positive for gibberellin, cytokinin, P solubilization, and siderophore production. In contrast, *B. aryabhattai* strain MoB09 produced 134.75 μg/ml of IAA and tested positive for siderophore production, phosphate solubilization, and ACC deaminase activity.

### Biodegradation of Paraquat in Liquid Medium

**FIGURE 4:**
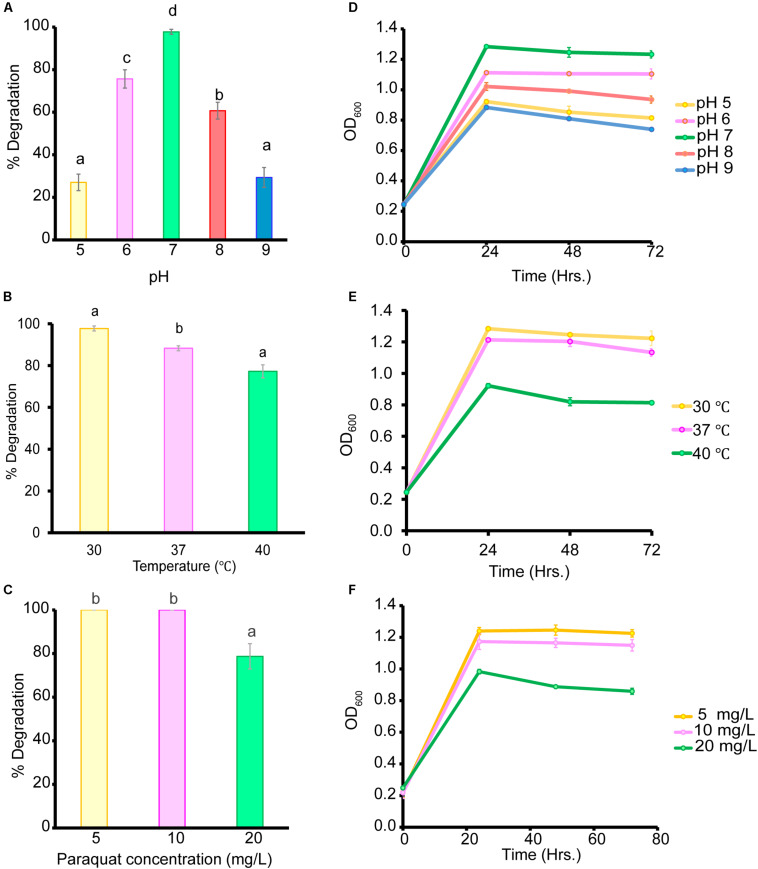
Effect of initial pH on paraquat degradation **(A)** and growth of bacteria **(D)**, cultivation temperature on paraquat degradation **(B)**, and growth of bacteria **(E)** and initial paraquat concentration on paraquat degradation **(C)** and growth of bacteria **(F)**.

**FIGURE 5:**
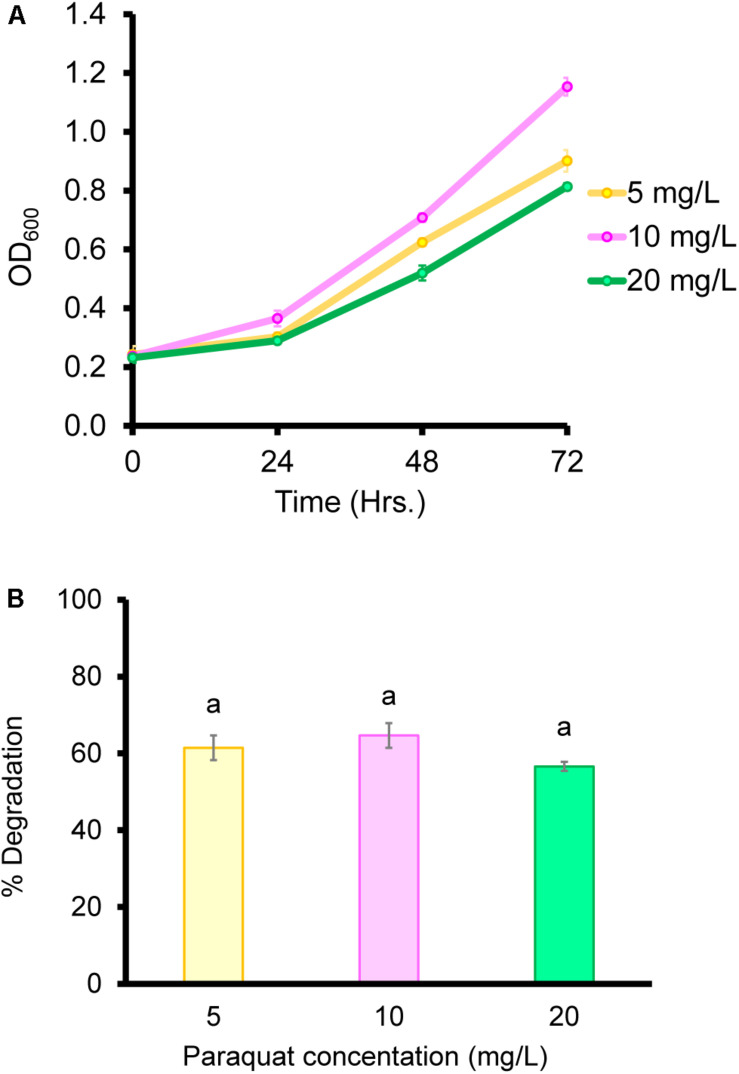
Growth of bacteria in paraquat as a sole carbon source and on paraquat degradation.

Biodegradation under controlled conditions is influenced by multiple factors such as temperature, pH, and pesticide concentration (Gangireddygari et al., 2017). In our study, the *B. aryabhattai* strain MoB09 showed maximum paraquat degradation at pH 7.0, 30°C and a paraquat concentration of less than 10 mg/L after 72 h of incubation (Figure 4). In addition, the *B. aryabhattai* strain MoB09 can utilize paraquat as a carbon source under controlled conditions (Figure 5).

Interestingly, some *Bacillus* spp. have been reported for their paraquat degradation. For instance, *B. cereus* was able to utilize 10 mg/L of paraquat as the sole carbon and nitrogen sources in synthetic media (Tu and Bollen, 1968). The *Bacillus altitudinis* strain XT12 can degrade 40.58% of 5 μg/ml methyl viologen (Binghua et al., 2015). The genetic level of paraquat resistance was evaluated in *B. subtilis*. The study revealed that in the case of the *sigM* gene, encoding for extra cytoplasmic function factors plays an important role in bacterial resistance toward paraquat oxidative stress (Cao et al., 2005).

The genus *Bacillus* is well known for its xenobiotic degradation and plant growth promotion abilities. For example, *B. megaterium* strains CCT 7729 and CCT 7730 were found to degrade 49.7 and 62.5% of mesotrione in 14 h (Dobrzanski et al., 2018), *B. thuringiensis* strain SG4 was found to degrade 85.0% of cypermethrin in 15 days (Pankaj et al., 2020) and 90% of chlorpyrifos 3,5,6-trichloro-2-pyridinol in 8 days (Samina et al., 2009). Our study supports the reports from those previous studies. In the controlled condition, *B. aryabhattai* strain MoB09 showed potential for paraquat degradation. This isolate could degrade almost 100% of 10 mg/ml of paraquat in 72 h. A previous study revealed that bacteria can degrade paraquat in two ways. First, they use paraquat as the sole nitrogen or carbon source, and second, they transform paraquat into low-toxicity or non-toxic products through co-metabolism (Huang et al., 2019).

Even though the *B. aryabhattai* strain MoB09 can grow in minimal media by using paraquat as its sole carbon source, growth and paraquat degradation were lower than that in NB. Since this strain was isolated by the molybdenum-reducing method, the paraquat degradation of this strain may have occurred via metabolic transformation, which would require extra electron donors such as glucose (Wu et al., 2013). The addition of glucose can enhance degradation of paraquat in *Achromobacter xylosoxidans* and *Streptomyces* sp. (Hashemi et al., 2015). The impact of paraquat concentration on the growth of organisms was also previously reported. They found that the biomass of the microbes was negatively correlated with paraquat concentration (Andy et al., 2015), which coincides with the results in this study. Paraquat showed toxic effects; the highest OD_600_ was obtained from the lowest paraquat concentration of 5 mg/L, and the lowest OD_600_ from the highest paraquat concentration of 20 mg/L. A higher paraquat concentration of 100 mg/L was also evaluated, but the *B. aryabhattai* strain MoB09 could not survive (data not shown). Although the metabolic product from paraquat degradation in this study was not evaluated, the metabolic pathways of paraquat in microorganisms have been suggested elsewhere (Huang et al., 2019). However, the paraquat metabolic pathways of *B. aryabhattai* strain MoB09 may be different from those found in that study, which is interesting and should be investigated in future works.

Application of paraquat-degrading bacteria with plant growth-promoting abilities is like killing two birds with one stone. The benefits are not only the resolution of the environmental concerns but also the enhancement of crop productivity, which is critical for non-agrochemical production. Nevertheless, the real challenge for the bioremediation process is the application of the isolated strain *in situ* under uncontrolled conditions. Accordingly, paraquat degradation and plant growth promotion of *B. aryabhattai* strain MoB09 were performed in cowpea under a pot experiment.

### Paraquat Degradation and Growth Promotion of Cowpea (*V. unguiculata*) Under Paraquat Stress

**FIGURE 6:**
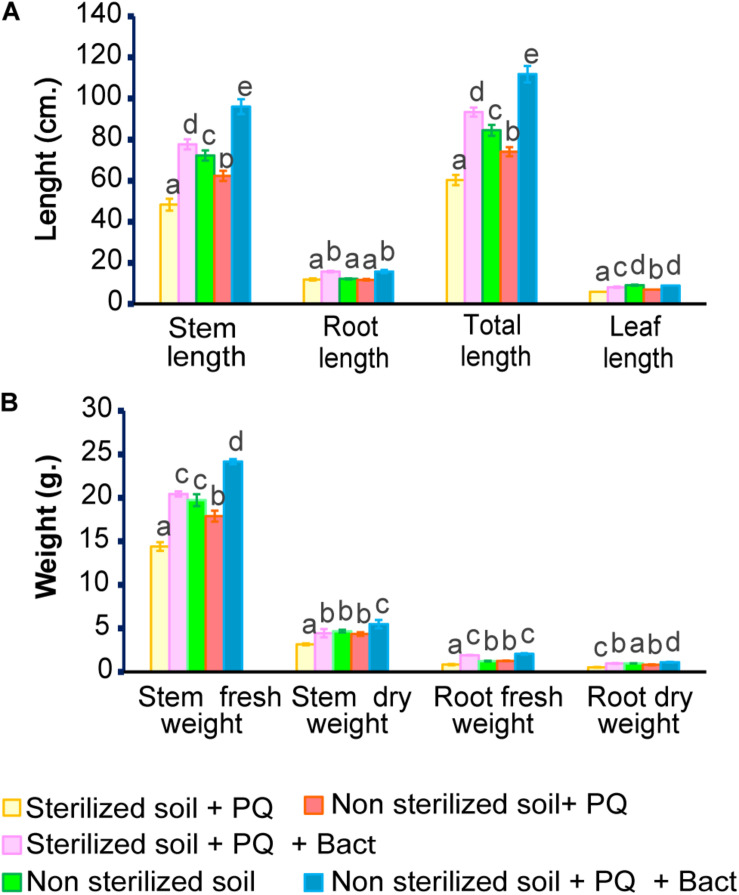
Growth parameters of cowpea (*V. unguiculata*) in soil with/without paraquat. **(A)** Stem, root, leaf and total length. **(B)** Fresh weight, dry weight of stem and root.

**FIGURE 7:**
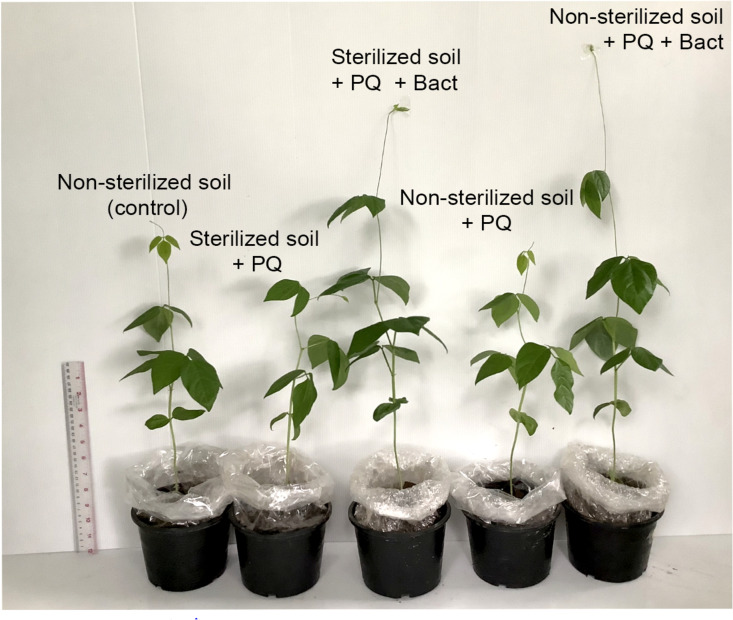
Growth of cowpea (*V. unguiculata*) (6 weeks age) in soil with/without paraquat in the pot experiment.

Cowpea (*V. unguiculata*) is sensitive to herbicide (Silva et al., 2020) and could serve as a model to perform the ability of bacterial isolates to promote plant growth under paraquat stress in pot experiments. The experiments were conducted in non-sterile and sterile soils. The properties of the soils used in this study are shown in Table 2. Cowpea seedlings inoculated with *B. aryabhattai* strain MoB09 significantly increased in stem length, root length, stem weight, and root weight in both sterilized and non-sterilized soils with paraquat (Figures 6, 7). In sterilized soil, *B. aryabhattai* strain MoB09 was the sole bacterium that promoted the growth of cowpea and remediated paraquat. In contrast, in non-sterilized soil with paraquat and *B. aryabhattai* strain MoB09, the growth parameters of cowpea, including fresh weight, dry weight, root length, total length, and leaf length, were higher than those in non-sterilized soil with paraquat only and non-sterilized soil alone (control). This result indicated that *B. aryabhattai* strain MoB09 may provide synergistic activities with other native microorganisms in the soil. However, the microbial community in the soil should be monitored in future works, in order to understand the population dynamic and survival of the inoculated bacteria.

**TABLE 2:** Soil properties used in the pot experiment.

**Property amount**	**Non-sterilized soil**	**Sterilized soil**
pH	7.16	7.14
Organic matter (%)	3.21	3.46
Total nitrogen (%)	0.14	0.15
Available phosphorus (mg/kg soil)	399.43	394.77
Exchangeable potassium (mg/kg soil)	244.14	250.34

The residues of paraquat in the soil ranged from 5 to 200 mg/kg. Therefore, the maximum value (200 mg/kg) was applied as the initial paraquat concentration in this study (FAO/WHO, 1971). Paraquat residue in soil was determined from the treatment of non-sterilized soil with paraquat and non-sterilized soil with paraquat and *B. aryabhattai* strain MoB09. It was found that 0.05 mg/kg soil of paraquat remains in non-sterilized soil with paraquat and *B. aryabhattai* strain MoB09, which was lower than that in non-sterilized soil without *B. aryabhattai* strain MoB09 (0.18 mg/kg soil).

### Growth Promotion of Cowpea (*V. unguiculata*) Under Drought Conditions in Soil by *B. aryabhattai*

**FIGURE 8:**
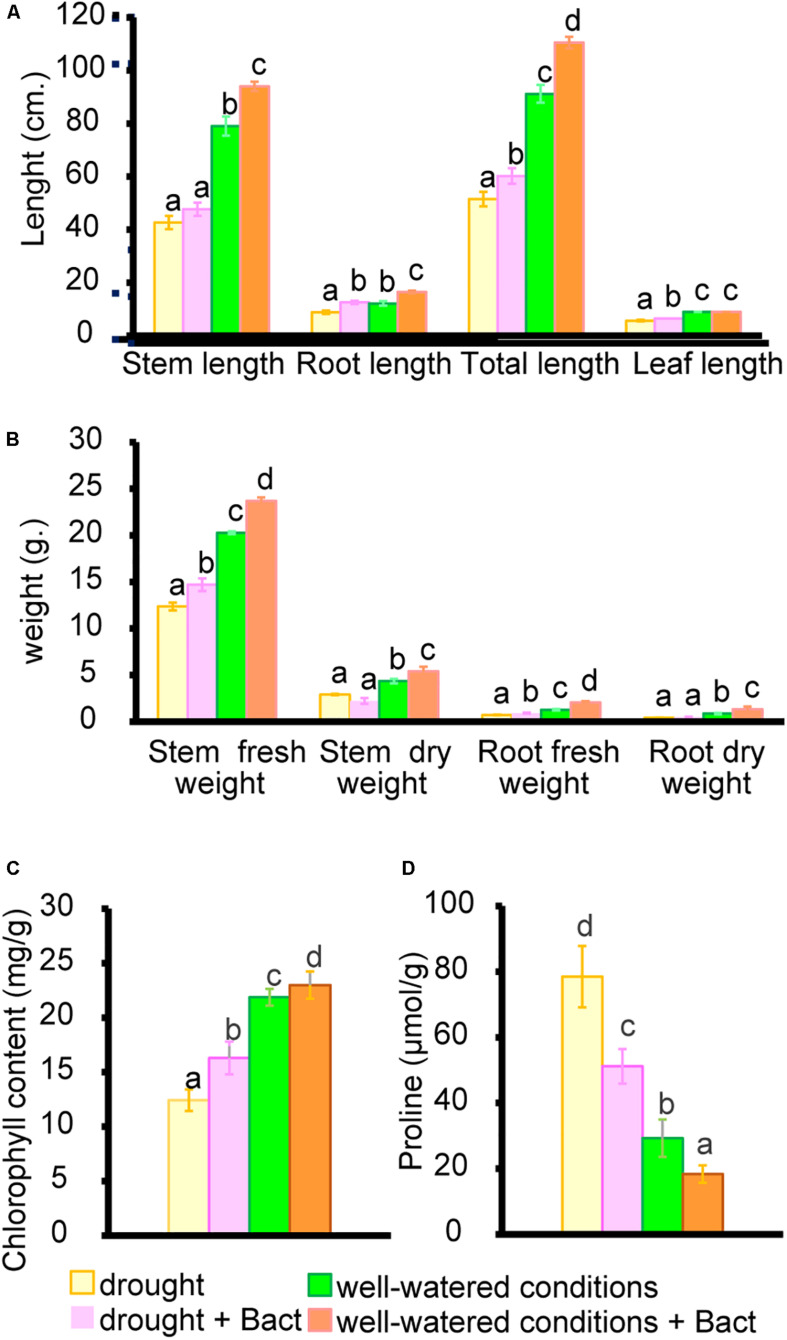
Growth parameters and proline content of cowpea (*V. unguiculata*) in well-watered and drought conditions with/without *B. aryabhattai* MoB09. **(A)** Stem root leaf and total length. **(B)** Fresh weight, dry weight of stem and root. **(C)** Chlorophyll contect and **(D)** Proline contents.

**FIGURE 9:**
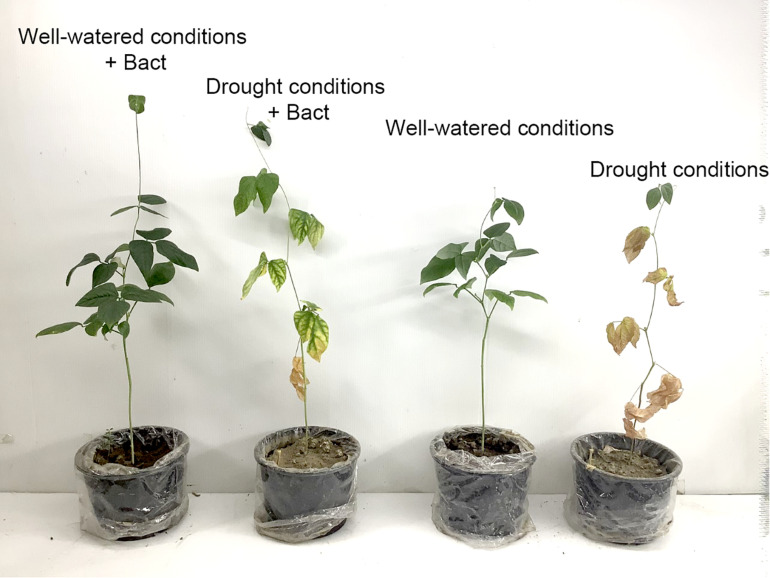
Growth of cowpea (*V. unguiculata*) in well-watered and drought conditions with/without *B. aryabhattai* MoB09 in day 45 after sowing.

Apart from residual pollutants remaining in the soil, environmental factors also generate various stresses, which affect plant growth and development. Environmental factors such as drought and salinity are of interest, as they affect the crop yield and quality of many plants, including cowpea (*V. unguiculata* L. Walp.). Cowpea is a suitable model for testing drought resistance due to its high sensitivity to water stress during flowering and pod-filling stages (Oyewole et al., 2017). Our study found that the completely withholding watering significantly suppresses various growth parameters, such as shoot length, root length, biomass weight, and chlorophyll content and enhances proline production (Figures 8, 9).

Our result agrees with reports from a previous study that drought stress affected all physiological parameters in plants, especially a significant decrease in chlorophyll a, chlorophyll b, and total chlorophyll contents (Mafakheri et al., 2010). Proline accumulation has been reported as a mitigation mechanism for drought stress in cowpea (Zegaoui et al., 2017). Enhancement in proline biosynthesis helps in balancing osmotic potential and maintaining cell turgor pressure (Chun et al., 2018).

*Bacillus* species such as *B. amyloliquefaciens* HYD-B17, *B. licheniformis* HYTAPB18, *B. thuringiensis* HYDGRFB19, and *B. subtilis* RMPB44, have been reported for their plant growth-promoting and positive effects on drought tolerance in maize (Vardharajula et al., 2011). *Bacillus* spp. have been applied in agriculture for a long time. Formulated and sporulated cultures of *B. thuringiensis* (Bt) are widely used for pest control (Crickmore, 2006). One of the advantages for the application of *Bacillus* spp. is their endospore-forming ability, which provides long-term stability and survival comparable with those of agrochemicals and other bacteria (Borriss, 2011).

## Conclusion

A great number of microorganisms have been reported for their paraquat degradation. However, there are hardly any bacteria capable of breaking down paraquat in soil, promoting plant growth, and enhancing drought tolerance altogether. Our results suggest that the *B. aryabhattai* MoB09 bacterium, which is screened from soil under heavy use of chemicals, can be very beneficial as a bioremediation agent in paraquat-contaminated sites and as a biofertilizer. It could promote the growth of cowpea (*V. unguiculata*) in soil under induced conditions of drought stress. The mechanisms of plant growth promotion and mitigation of drought stress as well as their plant growth-promoting hormone production will be evaluated in a future work. In addition, the application of this bacterium *in situ* will be conducted in a future study to remediate paraquat contamination in field trials.

## Data Availability Statement

The original contributions presented in the study are included in the article/Supplementary Material, further inquiries can be directed to the corresponding authors.

## Author Contributions

PI: conceptualization, methodology, formal analysis, investigation, and writing—original draft. PP: conceptualization, funding acquisition, writing—review and editing, and resources. JP: writing—review and editing. WP-A: writing—review and editing and resources. CP: supervision, conceptualization, project administration, funding acquisition, resources, and writing—review and editing.

## Conflict of Interest

The authors declare that the research was conducted in the absence of any commercial or financial relationships that could be construed as a potential conflict of interest.

## Footnotes

1 http://www.ncbi.nlm.nih.gov
